# Preventive Effect of Hydroxyzine on Postoperative Nausea and Vomiting: A Single-Center, Retrospective, Observational Cohort Study

**DOI:** 10.3390/jcm13247807

**Published:** 2024-12-20

**Authors:** Shosaburo Jotaki, Kenta Murotani, Teruyuki Hiraki

**Affiliations:** 1 Department of Anesthesiology, Kurume University School of Medicine, 67 Asahi-machi, Kurume 830-0011, Fukuoka, Japan; 2 Biostatistics Center, Kurume University, 67 Asahi-machi, Kurume 830-0011, Fukuoka, Japan; kmurotani@med.kurume-u.ac.jp

**Keywords:** hydroxyzine, postoperative nausea and vomiting, propensity score matching

## Abstract

**Background/Objectives:** Postoperative nausea and vomiting (PONV) is a common and distressing complication after surgery. Hydroxyzine, an affordable histamine H1 receptor antagonist with anxiolytic, moderate sedative, and antiemetic properties, is often used perioperatively; however, few studies have investigated its effect on PONV. In this study, we examined the efficacy of hydroxyzine in preventing PONV. **Methods:** This single-center, retrospective, observational cohort study included 647 female patients at risk of PONV between July 2021 and September 2022. The primary endpoint was PONV incidence on the day of surgery, and secondary endpoints included PONV incidence up to postoperative day 2 and emergence time, analyzed using propensity score matching. **Results:** The patients were categorized into two groups: 71 received hydroxyzine 25 mg (HYD group), and 576 received no prophylactic antiemetic treatment (NOT group). After adjustment for confounders, PONV incidence on the day of surgery was significantly lower in the HYD (n = 69) group compared to the NOT (n = 193) group (34.8% vs. 57.0%, *p* = 0.002), and similar results were observed up to postoperative day 2 (47.8% vs. 65.3%, *p* = 0.016). Emergence time did not differ between groups. **Conclusions:** Prophylactic administration of hydroxyzine could be effective in decreasing PONV incidence, though further randomized controlled trials are warranted to confirm these results.

## 1. Introduction

Postoperative nausea and vomiting (PONV) is a common post-surgical complication. PONV is an extremely distressing side effect and is associated with postoperative wound dehiscence and prolonged hospital stay. PONV incidence is 30% and can be as high as 80% in high-risk patients [1,2,3]. For these high-risk patients, guidelines recommend prophylactic administration of multiple antiemetics to reduce PONV incidence [4]. However, this strategy raises concerns about drug-related side effects [5], increased drug costs, and complexity in perioperative management. Moreover, the evidence base for some antiemetic agents remains insufficient, underscoring the need for further research [3,4].

Ondansetron, a 5-hydroxytryptamine type-3 antagonist, is the gold standard for PONV prevention worldwide and has recently been covered by insurance in Japan. Because of the limited number of drugs covered by health insurance in Japan, the use of this drug is expected to increase, although it remains relatively expensive (JPY 3386) [4,6,7]. Globally, rising healthcare costs are a major issue, particularly in Japan, owing to its aging population. Therefore, considering the cost-effectiveness of PONV preventive drugs is crucial [4,8].

Health insurance in Japan covers a few drugs, including hydroxyzine, metoclopramide, and ondansetron. Hydroxyzine, a histamine H1 receptor antagonist, is affordable (JPY 56) and possesses various effects, including anxiolytic effects, mild hypnotic effects, and the prevention of motion-sickness-induced emesis and adjunctive analgesia during labor [9,10,11,12,13]. Despite its widespread perioperative use, studies exploring hydroxyzine’s effects on PONV are limited [14,15]. In this study, we aimed to evaluate the prophylactic effects of hydroxyzine on PONV.

## 2. Materials and Methods

### 2.1. Patients and Study Design

This single-center, retrospective cohort study was conducted according to the guidelines of the Declaration of Helsinki and approved by the Ethics Committee of Kurume University Hospital (approval number: 22135; approval date: 12 December 2022). Per the guidelines of the Ethics Committee, informed consent was obtained from the patients using an opt-out approach. The inclusion criterion involved being at a higher risk of PONV, defined as either women aged 18–49 years who received inhaled general anesthesia or women aged ≥50 years who received inhaled general anesthesia and underwent surgery associated with a high risk of PONV, such as gynecologic or laparoscopic surgery and cholecystectomy [4]. Eligible patients admitted to hospital between 1 July 2021 and 30 September 2022 were included in the study. The follow-up period was 2 days after surgery. Data collection was between 12 December 2022 and 30 April 2023. Patient recruitment, follow-up, and data collection were conducted at Kurume University. Exclusion criteria included patients who underwent emergency surgery or neurosurgery, those with an American Society of Anesthesiologists physical status (ASA-PS) score of 4 or higher, surgeries lasting less than 30 min, those receiving intraoperative antiemetic drugs other than 25 mg hydroxyzine, those who returned to the hospital ward without extubation, patients discharged within postoperative day (POD) 2, patients who received antipsychotics on the day before surgery, patients without a nursing record for PONV, and those diagnosed with ileus within POD 2. Additionally, those who received opioid analgesics preoperatively were excluded because their perioperative course was considered unique, which could affect the efficacy of prophylactic antiemetic treatment [16,17].

### 2.2. Methodology

Patient data were collected from electronic medical and anesthesia records and analyzed after applying the inclusion and exclusion criteria. The patients were categorized into two groups: those who received only 25 mg hydroxyzine as prophylactic antiemetic treatment intraoperatively (HYD group) and those who received no prophylactic antiemetic treatment intraoperatively (NOT group). The primary outcome was PONV incidence on the day of surgery. The secondary outcomes included the incidence of PONV up to POD 2, use of rescue antiemetics up to POD 2, incidence of vomiting up to POD 2, emergence time, and incidence of severe adverse events such as new-onset arrhythmias or respiratory complications within POD 2. PONV was defined as the presence of nausea or vomiting or the administration of rescue antiemetics. Antiemetics used in the operating room included metoclopramide, ondansetron, hydroxyzine, or combinations of these, while antiemetic used in the hospital ward was metoclopramide. This information was captured in the electronic medical records. At our hospital, ward nurses assess PONV incidence every 1–2 h on the day of surgery and every 4–5 h from the day after surgery to the second day. When patients experienced nausea or vomiting, nurses or physicians asked them if they wished to receive antiemetic treatment. If the patient consented, antiemetics were administered; otherwise, the patient’s condition was monitored without intervention. The emergence time was defined as the time between the end of surgery and when the patient left the operating room. The collected data included: age; body mass index (BMI); ASA-PS score; postoperative opioid administration; smoking status (current- or non-smoker); type of surgery (abdominal, gynecological, head and neck, urological, orthopedic, superficial, and thoracic surgeries); laparoscopic surgery; surgery with a high risk of PONV; anesthesia and operation times; combined epidural anesthesia; anesthesiologist experience level (resident, fellow, or attending); intraoperative fentanyl, remifentanil, and ephedrine dose; intraoperative in–out balance; intraoperative sevoflurane and desflurane doses; type of inhalation anesthetic used (sevoflurane or desflurane); fasting time > 12 h; and postoperative steroid use. The smoking status was defined as “non-smoker” if the patient had quit smoking for at least 6 months prior to surgery. Head and neck surgery included procedures performed by otolaryngology and oral surgery departments, while superficial surgery included dermatology, plastic surgery, and breast surgery.

### 2.3. Anesthesia

Written informed consent for general anesthesia was obtained from all patients the day before surgery. General anesthesia was induced with remifentanil (0.15–0.2 mcg/kg/min), fentanyl (0.5–1 mcg/kg), propofol (1–2 mg/kg), and rocuronium (0.6–0.9 mg/kg). It was maintained with sevoflurane (1.5–2%) or desflurane (4–5%), remifentanil (0.1–0.3 mcg/kg/min), and a 50–60% oxygen/air mixture. Additional bolus administrations of fentanyl and rocuronium were used as required. All anesthetic drugs were discontinued at the end of the surgery, and residual muscle relaxation was reversed as required with sugammadex (2–4 mg/kg). Patients who had regular spontaneous breathing and obeyed commands were extubated and observed for approximately 5 min in the operation room. Subsequently, the patient was transferred to the hospital ward, as our hospital did not have a post-anesthesia care unit. The anesthesiologist in charge determined intraoperative antiemetic use and the choice of antiemetic.

### 2.4. Statistical Analysis

Categorical and continuous variables are presented as numbers (percentages) and medians (interquartile range), respectively. The categorical and continuous variables of the two groups were evaluated using the chi-squared and Wilcoxon rank sum tests, respectively. We performed 3:1 propensity score matching using the nearest neighbor method with a caliper width of 0.2 without replacement. The propensity score for the treatment group was calculated using a logistic regression model that included the following variables: age, BMI, ASA-PS scores (1 to 3), postoperative opioid administration (Yes, No), smoking status, type of surgery, laparoscopic surgery (Yes, No), surgery with a high risk of PONV (Yes, No), anesthesia and operation times, combined epidural anesthesia (Yes, No), anesthesiologist experience level (resident, fellow, or attending), intraoperative fentanyl, remifentanil and ephedrine dose, intraoperative in–out balance, intraoperative sevoflurane and desflurane doses, type of inhalation anesthetic used (sevoflurane, desflurane), fasting duration (fasting time < 12 h, >12 h), postoperative steroid use, less than 50 years old (Yes, No), and anesthesia time > 2 h (Yes, No). To assess performance, patient characteristics before and after propensity score matching were compared using a standardized mean difference of <0.1, which was considered negligible. We also employed sensitivity analysis using multivariate adjustment analysis. For the multivariate adjustment analysis, the following adjustment factors were selected based on the latest guidelines [4]: age, anesthesia time, postoperative opioid administration, smoking status, surgery with a high risk of PONV, ASA-PS scores, fasting duration, and anesthesiologist experience level. Statistical significance was set at *p* < 0.05. All statistical analyses were performed using the R statistical software version 4.2.2 (The R Foundation for Statistical Computing, Vienna, Austria; www.r-project.org).

## 3. Results

### 3.1. Baseline Participant Characteristics

**Figure 1:**
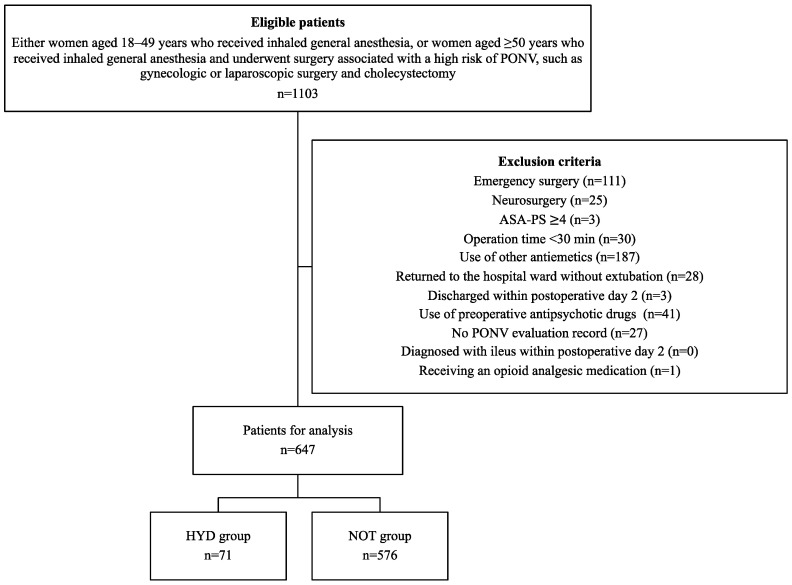
Flowchart for selecting patients for analysis. PONV, Postoperative nausea and vomiting; ASA-PS, American Society of Anesthesiologist physical status; HYD group indicates patients receiving 25 mg of hydroxyzine, and NOT group indicates those with no prophylactic antiemetic treatment.

Overall, 1103 patients were initially enrolled in this study. Of these, 647 patients (71 in the HYD group and 576 in the NOT group) were included in the final analysis after exclusion (Figure 1). Patients in the HYD group were younger, had longer operation times, and had a higher remifentanil dose than those in the NOT group (Table 1). A 3:1 propensity score matching analysis was employed to adjust for bias in patient characteristics. This resulted in all patient characteristics being adjusted, with standardized mean differences < 0.11 (Table 1).

### 3.2. Outcomes

The adjusted PONV incidence on the day of surgery and up to POD 2 was significantly different between the HYD and NOT groups, with rates of 34.8% vs. 57.0% (*p* = 0.002) and 47.8% vs. 65.3% (*p* = 0.016), respectively (Table 2). Furthermore, administration of rescue antiemetics up to POD 2 was significantly different between the HYD and NOT groups, with rates of 30.4% vs. 47.2% (*p* = 0.023), respectively (Table 2). However, no significant differences were found in the incidence of vomiting up to POD 2 and emergence time between two groups after adjustment (Table 2). The results of sensitivity analysis using multivariate adjustment were similar to those of the main analysis (Table 3). One patient in the HYD group experienced apnea at the time of discharge from the operating room. In contrast, no case of arrhythmias, such as QT prolongation or torsade de pointes (Tdp), was observed in the medical records of any patients within POD 2.

## 4. Discussion

In this study, hydroxyzine effectively prevented PONV in at-risk patients. Our results are significant because hydroxyzine has rarely been studied for preventing PONV. While previous studies have shown the efficacy of hydroxyzine in preventing PONV, they were conducted approximately 50 years ago and focused on intramuscular administration [14,15]. Intramuscular injection of hydroxyzine carries potential risks, including skin necrosis as reported in the literature [18,19], as well as nerve and muscle damage [20]. Given that intravenous access is routinely secured in the current operating room environment, intramuscular injection is rarely selected for drug administration. Our study is novel, demonstrating that hydroxyzine is effective in current clinical settings and with intravenous administration.

Hydroxyzine, a first-generation antihistamine, crosses the blood–brain barrier, enabling it to inhibit the action of histamine, a neurotransmitter in the central nervous system [7,11,17]. It exerts anxiolytic, sedative, and antiemetic effects by inhibiting the thalamus, hypothalamus, and limbic system [7,11,17]. The most studied first-generation antihistamine drug is dimenhydrinate [4], which has also been shown to prevent PONV [4,21]. However, hydroxyzine may have a longer-lasting effect because of its longer half-life of approximately 20 h, compared to dimenhydrinate’s of about 6 h [22,23].

Despite anticipating that hydroxyzine’s sedative side effect, drowsiness, might prolong emergence time, no difference was observed between the two groups. This can be due to several reasons. First, the patients in this study were young, making them more easily aroused from sedation. Second, the resultant drowsiness may have served as an appropriate form of sedation during the perioperative period, facilitating smooth extubation without agitation and delirium [11]. Although first-generation antihistamines have anticholinergic activity and can cause postoperative delirium [24], hydroxyzine, unlike other first-generation antihistamines, exhibits very weak anticholinergic activity [25,26]. In clinical practice, it is often used to treat patients with agitation and delirium [11,27]. Third, the timing of hydroxyzine administration in the HYD group, which was mostly at the start of wound closure, may have influenced the results. Hydroxyzine exhibits sedative effects that enhance the depth of anesthesia within 10 min after intravenous administration, lasting approximately 30–60 min [28,29]. The reason there was no difference in the emergence time may be because it usually takes more than 30–60 min from the start of wound closure to extubation.

In the HYD group, one patient experienced apnea, which occurred immediately after the administration of 30 mcg of fentanyl for postoperative pain. The interaction between fentanyl and hydroxyzine and the effect of high obesity (BMI = 36) are considered possible causes [30]. Although none of the patients in this study experienced new arrhythmias, it is worth noting that the United States Food and Drug Administration has indicated that QT prolongation syndrome or Tdp may occur with potentially linked to hydroxyzine use. The incidence of QT prolongation or Tdp associated with hydroxyzine has been reported as 3.81 cases per 1,000,000 patient-years of exposure [31]. All patients who developed QT prolongation or Tdp had at least one risk factor for such events, with most having multiple risk factors [31]. Therefore, hydroxyzine can be considered safe for use in patients without QT prolongation or Tdp risk factors. However, among patients with risk factors for QT prolongation or Tdp, hydroxyzine should be administered with caution.

In this study, the number of patients who received prophylactic antiemetic therapy was unexpectedly low despite the high risk of PONV. This may be owing to the fact that busy anesthesiologists do not consistently adhere to the guidelines [32,33]. Some anesthesiologists may have believed that intervention was only necessary once PONV occurred. Moreover, dexamethasone, an effective antiemetic, was not used intraoperatively in this study because it is not covered by insurance in Japan and is not stored in our operating room.

Further research is needed to explore the efficacy of hydroxyzine in preventing PONV, as many factors remain unclear, including the optimal dosage, timing of administration, and differences in efficacy depending on the type of surgery. Comparative studies with other antiemetic agents are also lacking. We propose conducting a randomized controlled trial (RCT) to compare hydroxyzine with ondansetron in terms of PONV prevention and cost-effectiveness. Since both ondansetron and hydroxyzine are covered by insurance in Japan, no ethical concerns will arise from conducting such an RCT.

### Limitations

This study had some limitations. First, we did not include data on the history of motion sickness or PONV, a known risk factor, as we do not customarily request these data during medical interviews at our hospital. Second, the timing of antiemetic administration could not be standardized because of the observational nature of the study. However, most patients in the HYD group were administered the medication at the time of wound closure. Third, there is a potential selection bias for patients in the HYD group, as hydroxyzine may have been administered to individuals less likely to experience delayed emergence. Therefore, it is difficult to generalize the results of this study to older patients with comorbidities, such as reduced renal function. Fourth, we acknowledged that the use of established PONV scales, such as the Rhodes Index of Nausea, Vomiting, and Retching [34], could have provided more nuanced insights into the severity and impact of PONV symptoms. However, in our clinical setting, routine documentation of PONV was limited to binary outcomes such as the presence or absence of nausea, vomiting, or use of rescue antiemetics. Finally, this was a single-center, retrospective study. Thus, there are external validity issues, and although we used propensity score analysis to adjust the perioperative patient characteristics, there are still biases due to unmeasured confounders and non-randomization. A multicenter randomized prospective study is required to validate our results.

## 5. Conclusions

In this study, the intravenous prophylactic administration of hydroxyzine could be effective in preventing PONV on the day of surgery in female patients at risk of PONV. However, careful consideration is required when using hydroxyzine in patients with risk factors for QT prolongation or in older patients with comorbidities. Further research, including well-designed randomized controlled trials, is warranted to clarify optimal dosing, administration timing, and its comparative efficacy against established antiemetics such as ondansetron.

## Figures and Tables

**Table 1:** Patient characteristics before and after 3:1 propensity score matching.

	Original Cohort		*p* Value	Matched Cohort		SMD
	HYD Group (n = 71)	NOT Group (n = 576)		HYD Group (n = 69)	NOT Group (n = 193)	
Age (years) *	46 [36, 61]	51 [39, 71]	0.023	46 [36, 62]	47 [36, 60]	0.017
BMI (kg/m^2^)	21.7 [19.1, 25.0]	22.0 [19.7, 25.0]	0.39	21.6 [19.1, 24.9]	21.9 [19.9, 24.9]	0.100
ASA-PS (%)						
1	36 (50.7)	221 (38.4)	0.061	35 (50.7)	100 (51.8)	0.022
2	33 (46.5)	309 (53.6)	0.31	32 (46.4)	89 (46.1)	0.005
3	2 (2.8)	46 (8.0)	0.18	2 (2.9)	4 (2.1)	0.053
Postoperative opioid administration (%)	23 (32.4)	132 (22.9)	0.11	22 (31.9)	54 (28.0)	0.085
Non-smoker (%)	67 (94.4)	529 (91.8)	0.61	65 (94.2)	177 (91.7)	0.098
Type of surgery (%)						
abdominal surgery	15 (21.1)	175 (30.4)	0.14	14 (20.3)	43 (22.3)	0.049
gynecological surgery	30 (42.3)	202 (35.1)	0.29	29 (42.0)	76 (39.4)	0.054
head and neck surgery	14 (19.7)	122 (21.2)	0.90	14 (20.3)	37 (19.2)	0.028
urological surgery	5 (7.0)	32 (5.6)	0.81	5 (7.2)	15 (7.8)	0.020
orthopedic surgery	2 (2.8)	12 (2.1)	1.00	2 (2.9)	4 (2.1)	0.053
superficial surgery	5 (7.0)	31 (5.4)	0.76	5 (7.2)	18 (9.3)	0.076
thoracic surgery	0 (0.0)	2 (0.3)	1.00	0 (0.0)	0 (0.0)	0.001
Laparoscopic surgery (%)	25 (35.2)	219 (38.0)	0.74	23 (33.3)	66 (34.2)	0.018
Risk surgery (%)	49 (69.0)	401 (69.6)	1.00	47 (68.1)	133 (68.9)	0.017
Anesthesia time (min)	282 [211, 384]	251 [174, 350]	0.054	278 [210, 378]	259 [190, 355]	0.007
Operation time (min)	216 [128, 310]	173 [106, 256]	0.035	214 [128, 292]	187 [120, 267]	0.006
Combined with epidural anesthesia (%)	31 (43.7)	204 (35.4)	0.22	38 (55.1)	104 (53.9)	0.024
Anesthesiologist (%)						
Resident	16 (22.5)	103 (17.9)	0.43	16 (23.2)	47 (24.4)	0.027
Fellow	39 (54.9)	310 (53.8)	0.96	39 (56.5)	99 (51.3)	0.105
Attending	16 (22.5)	163 (28.3)	0.38	14 (20.3)	47 (24.4)	0.098
Fentanyl dose (mL)	4.0 [2.0, 4.0]	3.0 [2.0, 4.0]	0.88	4.0 [2.0, 4.0]	4.0 [2.0, 4.0]	0.009
Remifentanil dose (mg)	2.7 [2.0, 4.0]	2.2 [1.5, 3.3]	0.014	2.6 [2.0, 3.8]	2.5 [1.8, 3.5]	0.027
Ephedrine dose (mg)	12 [7, 20]	12 [4, 20]	0.33	12 [6, 20]	12 [4, 22]	0.003
In-out balance (mL)	1390 [791, 1986]	1195 [569, 1747]	0.058	1340 [766, 1965]	1336 [590, 1879]	0.020
Sevoflurane dose (mL)	69.9 [45.5, 95.3]	60.4 [39.9, 89.6]	0.30	69.9 [45.8, 92.6]	63.0 [41.0, 89.9]	0.016
Desflurane dose (mL)	0.0 [0.0, 0.0]	0.0 [0.0, 0.0]	0.14	0.0 [0.0, 0.0]	0.0 [0.0, 0.0]	0.023
Desflurane use rate (%)	7 (9.9)	37 (6.4)	0.40	6 (8.7)	16 (8.3)	0.015
Fasting period >12 h (%)	18 (25.4)	161 (28.0)	0.75	18 (26.1)	49 (25.4)	0.016
Postoperative steroid use	2 (2.8)	11 (1.9)	0.95	2 (2.9)	4 (2.1)	0.053
Less than 50 years old (%)	42 (59.2)	278 (48.3)	0.11	41 (59.4)	113 (58.5)	0.018
Anesthesia time > 2 h (%)	69 (97.2)	527 (91.5)	0.15	67 (97.1)	188 (97.4)	0.019

* Data are expressed as number (%) or median [interquartile range]. HYD, hydroxyzine; NOT, no prophylactic antiemetic treatment; ASA-PS, American Society of Anesthesiologists physical status; BMI, body mass index; SMD, standardized mean difference.

**Table 2:** Outcomes before and after 3:1 propensity score matching.

	Original Cohort		*p* Value	Matched Cohort		*p* Value
	HYD Group (n = 71)	NOT Group (n = 576)		HYD Group (n = 69)	NOT Group (n = 193)	
PONV on the day of surgery (%) *	24 (33.8)	298 (51.7)	0.006	24 (34.8)	110 (57.0)	0.002
PONV up to POD 2 (%)	34 (47.9)	350 (60.8)	0.050	33 (47.8)	126 (65.3)	0.016
Use of rescue antiemetics up to POD 2 (%)	21 (29.6)	252 (43.8)	0.031	21 (30.4)	91 (47.2)	0.023
Vomiting up to POD 2 (%)	4 (5.6)	75 (13.0)	0.11	4 (5.8)	24 (12.4)	0.19
Emergence time (min)	34 [27, 41]	36 [28, 44]	0.38	35 [27, 41]	37 [28, 45]	0.37

* Data are expressed as number (%) or median [interquartile range]. HYD, hydroxyzine; NOT, no prophylactic antiemetic treatment; PONV, postoperative nausea and vomiting; POD, postoperative day.

**Table 3:** Outcomes adjusted for multivariate analysis for sensitivity analysis.

	NOT Group	HYD Group
PONV on the day of surgery *		
Adjusted odds ratio	1.00 (reference)	0.41 (0.24 to 0.69)
PONV up to POD 2		
Adjusted odds ratio	1.00 (reference)	0.50 (0.30 to 0.83)
Use of rescue antiemetics up to POD 2		
Adjusted odds ratio	1.00 (reference)	0.48 (0.27 to 0.82)
Vomiting up to POD 2		
Adjusted odds ratio	1.00 (reference)	0.42 (0.12 to 1.06)
Emergence time		
Adjusted regression coefficients	1.00 (reference)	−1.57 (−4.84 to 1.70)

* Data are expressed as adjusted odds ratios with a 95% confidence interval and adjusted regression coefficients with a 95% confidence interval. Multivariate adjustment was performed with age, anesthesia time, postoperative opioid administration (Yes, No), smoking status, surgery with a high risk of PONV (Yes, No), ASA-PS scores (1 to 3), length of fasting period (fasting time < 12 h, more than 12 h), and level of anesthesiologist experience (resident, fellow, attending). HYD, hydroxyzine; NOT, no prophylactic antiemetic treatment; PONV, postoperative nausea and vomiting; POD, postoperative day.

## Data Availability

The data that support the findings of this study are available from the corresponding author, S.J., on reasonable request.
